# Two Subgroups of Porcine Circovirus 2 Appearing among Pigs in Southern China

**DOI:** 10.1128/genomeA.00942-14

**Published:** 2014-09-25

**Authors:** Minxiu Zhang, Zhixun Xie, Liji Xie, Xianwen Deng, Zhiqin Xie, Sisi Luo, Li Huang, Jiaoling Huang, Tingting Zeng

**Affiliations:** Guangxi Key Laboratory of Animal Vaccines and Diagnostics, Guangxi Veterinary Research Institute, Nanning, Guangxi, China

## Abstract

We report here two complete genome sequences of porcine circovirus 2. The complete genomes of BH5 and BH6 were amplified and analyzed. Sequence analysis demonstrated that strains BH5 and BH6 belonged to PCV2d and PCV2b, respectively. Knowledge regarding the complete genome sequences of strains BH5 and BH6 will be useful for epidemiological surveillance.

## GENOME ANNOUNCEMENT

Porcine circovirus 2(PCV-2) belongs to the *Circoviridae* family, which is a small nonenveloped virus and contains a single-stranded circular DNA genome of about 1.76 kb (1, 2). The genome contains two open reading frames, including ORF1 and ORF2 (3). ORF1 consists of 945 nucleotides (nt), encoding 314 amino acids, called the Rap protein, responsible for viral replication proteins. ORF2 consists of 702 or 705 nt, encoding 234 or 235 amino acids, called the Cap protein (4). Porcine circovirus 2 (PCV-2) is considered to be the main pathogen that causes postweaning multisystemic wasting syndrome (PMWS), an epidemic on pig farms (5). PCV-2 has serious economic impact on the pig industry. Thus, it is important to enhance the surveillance of PCV-2.

In this study, various tissue samples (liver, spleen, tonsil, lymph nodes) were collected from pigs at a farm in Guangxi, China. DNA was extracted from various tissue samples with a viral DNA/RNA kit (TransGen, Beijing, China). The complete genomes of PCV-2 strains BH5 and BH6, which were collected from the same pig farm, were ampliﬁed by PCR using Primers (6). The ampliﬁed products were puriﬁed and cloned into the pMD-18T vector (TaKaRa) and sequenced (TaKaRa, Dalian, China) (7–9). Sequences were assembled and manually edited to generate the ﬁnal full-length genome sequences (10–14).

The genomes of strains BH5 and BH6 consist of 1,767 nt and the ORF1 of strains BH5 and BH6 consists of 945 nt, encoding 314 amino acids. ORF2 of strain BH5 consists of 705 nt, encoding 235 amino acids; ORF2 of strain BH6 consists of 702 nt, encoding 234 amino acids. The genome sequence of strain BH5 presented 96.4% similarity compared with the strain BH6. Genome sequences of strain BH5 shared the highest sequence homology (98.8%) with porcine circovirus 2 isolate Henan (GenBank accession no. AY969004). Genome sequences of strain BH6 shared the highest sequence homology (97.9%) with porcine circovirus type 2 strain TJ (GenBank accession no. AY181946). The homologies of strain BH5 with the sequences of representative porcine circovirus 2 strain BF and France (GenBank accession no. AF381175 and AF055394) were 95.2% and 96.2%, and the homologies of strain BH6 with these strains were 96.2% and 98.7%.

PCV2 can be divided into five subgroups according to ORF2. These five subgroups are PCV2a, PCV2b, PCV2c, PCV2d, and PCV2e. Phylogenetic analysis showed that strains BH5 and BH6 belong to PCV2d and PCV2b. The results showed that strain BH6 has a close relationship with porcine circovirus 2 isolate Henan, and also has a relationship with the porcine circovirus 2 isolate DK1987PMWSfree (GenBank accession no. EU148504). Strain BH5 has a close relationship with porcine circovirus 2 isolate TJ06 (GenBank accession number EF524539), and also has a relationship with the porcine circovirus 2 isolate DK1987PMWSfree. These results suggested two subgroups of porcine circovirus 2 exist in the same pig farm and showed some degree of complexity in the epidemic of porcine circovirus 2.

### Nucleotide sequence accession numbers.

The complete genome sequences of BH5 and BH6 were deposited in GenBank under the accession numbers KJ956689, KJ956690, KJ956691, KJ956692, and KM245558.
